# Understanding and measuring the work‐related quality of life among those working in adult social care: A scoping review

**DOI:** 10.1111/hsc.13718

**Published:** 2022-01-23

**Authors:** Barbora Silarova, Nadia Brookes, Sinead Palmer, Ann‐Marie Towers, Shereen Hussein

**Affiliations:** ^1^ Personal Social Services Research Unit University of Kent Cornwallis Central Canterbury UK; ^2^ Centre For Health Services Studies George Allen Wing Cornwallis Building University of Kent Canterbury UK; ^3^ Present address: Centre For Health Services Studies George Allen Wing Cornwallis Building University of Kent Canterbury CT2 7NF UK; ^4^ Present address: Faculty of Public Health Policy London School of Hygiene & Tropical Medicine London WC1E 7HT UK

**Keywords:** care staff, care worker, carer, employee, job‐related well‐being, support worker, work‐related wellbeing

## Abstract

The main aim of this scoping review was to understand how work‐related quality of life (WRQoL) in adult social care has been defined and measured in the literature and to map key components of WRQoL among those working in adult social care and similar contexts. The scoping review included studies that: 1‐ focused on WRQoL/work‐related wellbeing (and their synonyms); and 2‐ included adult social care or community health care. We searched existing evidence from November 2019 until July 2020 through an electronic literature search of eight major databases complemented by the grey literature, searching the reference lists and by contacting our existing network of experts in the field. In addition, we repeated the searches to identify any relevant literature published in 2021. Reporting followed the PRISMA Extension for Scoping Reviews (PRISMA‐ScR) checklist. In total, we included 68 publications. These publications indicate that there is an absence of agreement on a definition and measurement of WRQoL in adult social care. Based on a thematic analysis we identified six key components of WRQoL: organisational characteristics; job characteristics; mental wellbeing and health; physical wellbeing and health; spill‐over from work to home; and professional identity. In summary, at the moment, there is no agreement on what WRQoL is and how to measure it in adult social care. As a result, there is very limited evidence on how to improve WRQoL among people working in adult social care. However, this scoping review suggests that there are six key components of WRQoL that researchers may consider to include in their future studies.

**What is known about this topic**

- Adult social care in England has been experiencing a workforce crisis.
- Work‐related quality of life (WRQoL) is one of the factors that may help us to understand how adult social care can become an attractive sector to work in.
- A comprehensive evidence synthesis of understanding WRQoL across different adult social care settings and workforce is lacking.

**What the paper adds**

- There is an absence of agreement on the definition of WRQoL in adult social care and how to measure it.
- We have identified seven measures of WRQoL used in adult social care.
- Based on the evidence we conclude that WRQoL in adult social care is a complex concept including six key components.

## 1 INTRODUCTION

Adult social care in England “covers social work, personal care and practical support for adults with a physical disability, a learning disability, or physical or mental illness, as well as support for their carers” (p4, (National Audit Office, 2018)) and it has been experiencing many long‐standing challenges, which have been exacerbated during the COVID‐19 pandemic (Bottery, 2020). These include, for example, limited access to publicly funded adult social care services, a fragmented social care market (services provided through local authorities, for‐profit and voluntary home care and residential care providers and directly employed individuals), demographic trends (e.g. people are living longer with multiple or complex needs and therefore requiring more social care services) and a workforce crisis including the inability to attract and retain a sufficient supply of care workers (Bottery et al., 2018; Skills for Care, 2020; The King's Fund, 2019). It has been estimated that in 2019/2020, 1.52 million people worked in adult social care in England with a vacancy rate of 7.3% and turnover rate of 30.4% for the 12 month period (Skills for Care, 2020). Given the projections that increasing numbers of people will require some form of social care support over the next decades (Bottery et al., 2018), it is essential to understand how to ensure that the adult social care sector can become an attractive and rewarding sector to work in.

Work‐related quality of life (WRQoL) has become an important concept internationally across different professions (Brauchli et al., 2017; Mokarami et al., 2016; Somsila et al., 2015; Vander Elst et al., 2012), including adult social care (André et al., 2014; Arts et al., 2001; Eustis et al., 1993b; Vermeerbergen et al., 2017) and is one of the factors associated with higher retention rates (Ricardo Rodrigues and Leichsenring, 2018; Scanlan et al., 2013). In addition, research shows that WRQoL is associated with the individual's physical and emotional wellbeing and with organisational and work outcomes, such as turnover and quality of work (Carr, 2014; Edwards et al., 2000; Maben, 2012). More importantly, in adult social care, similar to health care, WRQoL is linked to the quality of services provided and outcomes related to patients and service users (Boorman, 2009; Carr, 2014; Maben, 2012; Paparella, 2015). While several studies examined WRQoL in adult social care and similar settings (André et al., 2014; Arts et al., 2001; Eustis et al., 1993a; Vermeerbergen et al., 2017) there is no agreement on what WRQoL is, and in turn how this concept should be measured (Rai, 2015). The lack of consensus on both the definitions and measurement raises several challenges, including an inability to compare and synthesise quantitative evidence on WRQoL. Without a clear understanding of what WRQoL is and how to measure it, development of any interventions and recommendations to the sector and policymakers on how to improve WRQoL among those working in adult social care remains challenging.

The existing international evidence syntheses in social care only focused on a subset of adult social care settings and workforce such as nursing homes (André et al., 2014; Vermeerbergen et al., 2017) and home help services (Arts et al., 2001; Eustis et al., 1993a). As such, a comprehensive evidence synthesis covering different aspects of adult social care settings and workforce is lacking. The main objective of the present study was to understand how WRQoL has been defined and measured in the literature and to map critical components of WRQoL among those working in adult social care and other similar contexts. More specifically, we aimed to answer the following research questions: 1. What are the existing definitions of WRQoL among those working in adult social care; 2. What are the main components of WRQoL among this group of workers; 3. What aspects of adult social care work have an impact on the social care worker's quality of life; 4. What questionnaires of WRQoL are available to be used among those working in adult social care; 5. What strategies have been implemented and evaluated in adult social care that addressed social care staff WRQoL?

## 2 METHODS AND ANALYSIS

We conducted the scoping review following the updated guidance by the Joanna Briggs Institute (Peters et al., 2017) and a pre‐defined study protocol (available from the corresponding author). Reporting followed the PRISMA Extension for Scoping Reviews (PRISMA‐ScR) checklist (Tricco et al., 2018).

### 2.1 Eligibility criteria

The full eligibility criteria are described in Table S1. We included studies that: 1‐ focused on WRQoL/work‐related wellbeing (and their synonyms) where the concept is defined as a multidimensional construct consisting of several components (at least two); and 2‐ included adult social care or community health care and individuals working in those contexts as participant groups (such as direct care workers, managers or supervisors, occupational therapists, safeguarding and review officers, nurses, nursing aides, nursing and health care assistants, other allied health care professionals and registered professionals). As our preliminary search did not return a sufficient number of results when focusing only on adult social care context, we expanded our search to include community health care settings. The search expansion was to ensure we were able to understand what constitutes key components of WRQoL among people who work in adult social care by learning from other professions that are as close as possible to those working in social care settings.

### 2.2 Search strategy

We developed the search strategy with input from a research librarian from the Academic Liaison Services, Information Services, University of Kent. We searched existing evidence from November 2019 until July 2020. We performed an initial electronic literature search of three major databases (PubMed; CINAHL Plus with Full Text through EBSCO and Social Care Online) followed by an analysis of the title and abstract of retrieved papers, and of the index terms used to describe the articles to check whether our search strategy needed any refinement. As a next step, we replicated our literature search across an additional five databases (APA PsychINFO through EBSCO; Web of Science Core collection; Cochrane Library; Abstracts in Social Gerontology through EBSCO; Social Policy and Practice through Ovid). The grey literature was searched through the following databases: PROSPERO; OpenGrey; EThOS e‐theses online service; and ProQuest Dissertations & Theses Global. In addition, we repeated the searches as described above to identify any relevant evidence published in 2021 (until September including). We complemented these electronic searches by searching the reference lists of included full‐text reports and articles. Additionally, we contacted our existing network of 15 international experts to identify unpublished studies and interim findings from ongoing research. We did not contact authors of primary studies for further information as this was not required.

Examples of search strategies for electronic databases are included as Table S2.

### 2.3 Study selection

All the citations identified were downloaded into EndNote. Duplicates were auto‐removed in a combined library. Study selection was a challenging task as the social care sector has changed over time and varies between different countries including the use of terminology. To ensure that all relevant studies were correctly included, at the title and abstract identification level, only studies clearly not relevant were excluded. Each paper assessed as a full text was screened by at least two reviewers. To be more specific, first, titles and abstracts of the complete list of identified papers were divided equally and assessed by BS and NB. Both authors then met on several occasions to discuss all studies that were selected for inclusion as well as publications where the decision was unclear. The inclusion of studies for the full‐text stage was agreed during the discussions, and those not relevant were rejected. For publications where a decision to exclude could not be reached based on the title and abstract alone, the full paper was obtained for detailed assessment. Next, at least two reviewers BS and NB/SP independently assessed the full‐text articles for inclusion in the scoping review. Papers identified as not meeting the inclusion criteria by two researchers were excluded. Another researcher (SH/A‐M T) reviewed any papers where an agreement was not reached between BS, NB and SP. Reviewers were not masked to the author or journal name.

### 2.4 Data extraction/charting the results

A data extraction table (available from the corresponding author) was developed and included details on a) Study citation; b) Country; c) Study design; d) Context (e.g. domiciliary care, care home, nursing home); d) Participants (recruitment, inclusion criteria, age, sex, number, type of participant: e.g. personal assistant, care assistant, care worker, care staff, support worker); e) Results (disciplinary tradition/reference to the theoretical framework/model; definition of WRQoL; components of WRQoL and their definitions; measures of WRQoL; dependent variables associated with WRQoL; variables measured as independent variables associated with WRQoL; mechanisms of improving WRQoL). All data were extracted by BS, 10% checked for accuracy by NB and another 10% by SP.

### 2.5 Quality of studies

We did not assess the quality of studies included in the scoping review.

### 2.6 Presentation of results

We present the results in Tables and Figures to describe the key characteristics of the publications included and key components of WRQoL. Where possible, we conducted a narrative synthesis using thematic analysis to summarise information following Popay's framework (Popay et al., 2006). First, the key definitions of WRQoL, its components and measures were tabulated and combined into groups. After a process of familiarisation with the data, each group was qualitatively coded by one reviewer (BS) and discussed with NB and SP. These coded groups then formed preliminary themes/key components of WRQoL that were reviewed by BS, NB and SP and agreed upon.

## 3 RESULTS

### 3.1 Selection of sources of evidence

**FIGURE 1:**
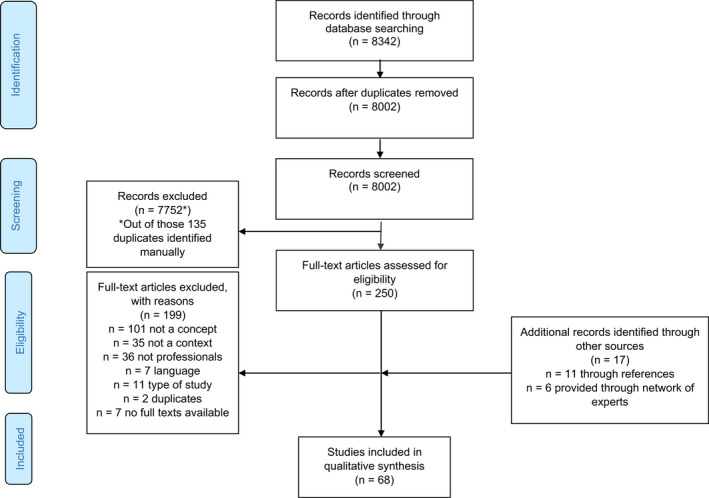
Prisma flow diagram

The PRISMA diagram (Figure 1) summarises the total number of studies identified and screened, assessed for eligibility, and included in the review, with reasons for exclusions. After removing duplicates across all electronic databases and grey literature, we (BS and NB) identified and assessed at the title and abstract level 8002 records. Out of those, we (BS, NB and SP) evaluated 250 full‐text articles for eligibility and included 51 studies. We identified 17 further papers through reference lists of eligible full‐text articles and our network of experts. In total, we included 68 publications in the current qualitative synthesis.

### 3.2 Characteristics of sources of evidence

Table 1 contains information on study citation, country, study design, context and type of participants. Almost half of the studies (30) included participants living in continental Europe, 12 studies were conducted in the USA and nine in Canada. Eight studies were conducted in the UK or England, three in Australia, two in Israel, one in South Africa, Taiwan and Japan respectively (studies included in the evidence synthesis are not included in this description). The type of participants and context varied across studies representing the diversity and complexity of professions and areas of adult social and community health care.

**TABLE 1:** Characteristics of sources of evidence and identified key areas of work‐related quality of life they reflect

	Study citation	Country	Study design	Context	Type of participant	Measures of WRQoL	OC	JC	MWaH	PWaH	S	PI
Studies identified through database searching												
1	André et al. (2014)	Articles from the USA and Canada	Evidence synthesis	Nursing homes	Administrators; ombudsmen; department staff, different kinds of healthcare workers	Not applicable	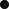					
2	Arts et al. (2001)	Origin of studies included not reported	Evidence synthesis	Domiciliary care	Home help aides	Not applicable	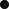	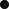	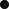	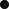		
3	Arts et al. (1999)	The Netherlands	Cross‐sectional	Domiciliary care	Home helps	***Workload*:** 'Experience and Assessment of Work' (van Veldhoven & Meijman, 1994) ***Psychological and physical outcomes*:** *job satisfaction*: from Boumans (Boumans, 1990) *burnout*: the Dutch translation of the Maslach Burnout Inventory (MBI‐NL) (Wilmar Schaufeli & van Dierendonck, 1994) *health:* a self‐assessment of general health ***Capacity for coping:*** *social support*: from the Organizational Stress Questionnaire (VOS‐D) (Bergers et al., 1986) *leadership style scale*: by Boumans (Boumans, 1990) based on the Algera (Algera, 1981) *ways of dealing with problems*: shortened version (van Dierendonck et al., 1992) of the Utrecht Coping List (UCL) (Scheurs et al., 1988)	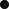	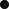	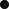	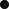		
4	Berendonk et al. (2019)	Germany	Cluster‐randomised controlled trial	Long‐term care facilities	Registered nurses; health care aides; nursing students; other	***Job demands and psychological work strain***: BHD (Hacker & Reinhold, 1999) ***Workplace conditions***: modified version of the Task and Job Analysis Tool – Residential LTC Version (TAA‐A) (Bussing & Glaser, 2002)		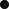	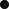			
5	Berta et al. (2018)	Canada	Cross sectional	Long‐term and community care	Health support workers	***Quality of Work Life:*** Quality of Work Life Measure (13 items) (Sirgy et al., 2001)		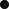			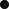	
6	Brousseau et al. (2008)	Canada	Descriptive phenomenological	Community‐based health and social service centres	Nurses	***Quality of working life*** : semi‐directed individual interviews	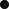	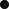	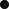		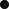	
7	NACAS (2018)	Not reported	Cross sectional	Not reported	Care worker; support worker; healthcare assistant; healthcare support worker; field supervisor; care coordinator; care manager; other	***Mental Health & Well‐being*** (11 questions) ***Physical Well‐being*** (10 questions) ***Economic Well‐being*** (7 questions) ***Perceptions of care work*** (5 questions) ***Other questions*** (9 questions)	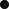		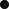	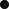		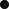
8	Catanzaro (1992)	The USA	Thesis: pre‐post quasi experimental	Nursing homes	Nursing aides	***Perceived influence:*** adaptation of scale by Vroom (Vroom, 1959) ***Satisfaction with influence:*** as used by Rafaeli (Rafaeli, 1989) ***Organisational commitment:*** Organizational Commitment questionnaire (Mowday et al., 1979) ***Turnover intention:*** from the Michigan Organizational Assessment Questionnaire (Cammann et al., 1983) ***Role conflict and role ambiguity:*** as used by Rizzo (Rizzo et al., 1970) ***Satisfaction of higher order needs:*** need satisfaction scale (Lawler & Hall, 1970) ***Satisfaction with service role:*** the Minnesota Satisfaction Questionnaire social service scale (Weiss et al., 1967) ***Satisfaction with organisational policies:*** the Minnesota Satisfaction Questionnaire social service scale (Weiss et al., 1967)	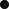	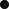	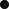			
9	Chamberlain et al. (2019)	Canada	Cross sectional (results only extracted from this part of the study)	Nursing homes	Care aides	***Care Aide Work Life*** ***job satisfaction*** *:* the Michigan Organizational Assessment Questionnaire Job Satisfaction Subscale (MOAQ‐JSS−3) (Ginsburg et al., 2016) ***Burnout:*** the Maslach Burnout Inventory (MBI) 9‐item Short Form (Maslach & Jackson, 1981) ***Experiences of dementia related responsive behaviours from residents:*** six questions		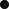	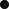			
10	Chou et al. (2011)	Taiwan	Cross sectional	Domiciliary care	Home care worker	***Quality of life*** : the WHOQOL‐BREF Taiwan version (Yao et al., 2002)	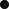		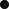	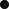		
11	Coffey et al. (2009)	The UK	Mixed method: Phase 1: interviews and open questions in the questionnaire Phase 2: focus groups	Social Services Department	Staff from Children & Families Division; Adult Older Division, Directorate and Support Services Division; Adult People with Special Needs Division	Open questions in the questionnaire, in‐depth interviews and focus groups, and measure of well‐being (the GHQ‐12) (Mullarkey et al., 1999)	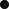	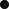	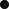			
12	Durkin et al. (2016)	England	Cross sectional	Community student nurses	Registered community nurses	***Work‐related quality of life*** : the Professional Quality of Life (ProQOL) Scale (B. Hudnall Stamm, 2009)			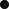			
13	Flynn et al. (2018)	England and Wales	Cross sectional	Residential care homes and supported living services	Managers; support workers; assistant psychologist	***Burnout:*** Maslach Burnout Inventory – human services version (MBI) (Maslach et al., 1996) ***Staff empathy for people with challenging behaviour***: the Staff Empathy for People with Challenging Behaviour Questionnaire (SECBQ) (Hutchinson et al., 2014) ***Challenging behaviour self‐efficacy***: the Challenging Behaviour Self‐efficacy Scale (CBSE) (Hastings & Brown, 2002) ***Staff positive contributions:*** the short version (Lunsky et al., 2014) of the Staff Positive Contributions Questionnaire (Hastings & Horne, 2004)	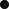	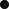	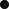			
14	Fortune (2006)	Canada	Thesis: qualitative	the Health Care Team in Aging and Veterans’ Care	Frontline staff from physiotherapy, occupational therapy, recreation therapy, music therapy, audiology, social work, nursing, clinical nutrition, and chaplaincy	In‐depth interviews and participant's observation focusing on quality of work life	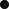	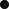	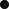			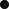
15	Hodgkins et al. (2005)	England	Pre‐post longitudinal	Residential site containing bungalows	Staff working at residential site for people with learning disabilities	***Strain, demands and support***: the Support and demands questionnaire (Rose, 1999) and the Support staff questionnaire (Harris & Rose, 2002) ***The perception of the team***: the Team climate inventory (Anderson & West, 1994) ***Burnout:*** the Maslach burnout inventory (Maslach et al., 1996) ***Anxiety***: a short scale measuring anxiety	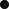	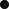	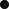			
16	Iecovich (2011)	Israel	Cross sectional	Domiciliary care	Migrant live‐in workers	***Work psychosocial characteristics:*** 30 item questionnaire (Pikhart et al., 2001)	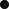	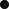				
17	Itzick et al. (2018)	Israel	Cross sectional	Welfare, health, community and correctional social care	Social workers	***Professional quality of life:*** the Professional Quality of Life Scale—Revised (ProQOL)(B. Hudnall Stamm, 2005)			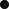			
18	Josefsson et al. (2011)	Sweden	Cross sectional	Special housing for older people	Registered nurses	A structured questionnaire designed for this study, focused on ***what the good work implies for registered nurses***. The questions were from two questionnaires (Anderson & West, 1994; Hagström et al., 1996)	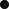	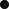				
19	Kemper et al. (2008)	The USA	Cross sectional	Skilled nursing and assisted living facilities; home care agencies	Direct care workers	Open ended question in questionnaire: What is the single most important thing your employer could do to improve your job as a direct care worker?	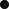	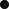				
20	Laurence (1993)	Canada	Cross sectional	Nursing homes; homes for the aged; rest and retirement homes; chronic care units; extended care	Staff working at long term care facilities	**Job preferences**: would you prefer a job elsewhere and why?	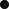	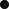	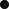			
21	MacEachron et al. (1985)	The USA	Randomised controlled trial	Residential services for people with intellectual disabilities at a large state school	Staff	***The job design characteristics***: the Job Diagnostic Survey (J. R. Hackman & Oldham, 1974) ***Influence in decision‐making:*** the actual level of participation in decision‐making; the slope of influence in decision‐making; total amount of control ***The leadership style:*** the Ohio State Leader Behavior Description Questionnaire (Halpin & Winer, 1957)	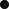	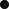				
22	Malherbe & Hendriks (2004)	South Africa	Mixed method: overview of American, British and South African literature supported by survey	The welfare organisation	Grassroots social workers	***Components of quality of work life:*** to indicate if different components are important to them regarding good quality of work life. ***Programmes for the improvement of quality of work life***: to indicate which programmes they believed would make the greatest contribution to the improvement of quality of work life in the organisation.	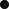	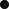			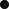	
23	Mänttäri‐van der Kuip (2014)	Finland	Cross sectional	Statutory social services for families and/or adults	Social workers	***Experiences of impaired subjective work‐related well‐being*:** a 7‐point Likert‐scale comprising five variables			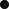		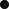	
24	McConachie et al. (2014)	The UK	Longitudinal between‐within subjects design	Care organisations	Support staff involved in the direct care of individuals with intellectual disabilities	***Psychological well‐being:*** the Warwick‐Edinburgh Mental Well‐Being Scale (WEMWBS) (Tennant et al., 2007)	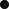		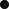			
25	Munir et al. (2012)	Denmark	Longitudinal	Elderly care	Health‐care assistants, nurses, physiotherapists, cleaning personnel, canteen personnel and maintenance staff	***Psychological wellbeing:*** 5 items (Bech et al., 2003)	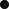		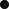		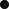	
26	Pătraș et al. (2018)	Spain	Cross sectional	Organisations offering services to people with intellectual disability	Primary health care workers, psychologists, occupational therapists, social workers and physiotherapists	***Contribution‐to‐others wellbeing beliefs*** : the Beliefs about Wellbeing Scale (McMahan & Estes, 2011), adapted to the workplace (Pătraş et al., 2017)	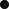		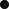			
27	Perreira et al. (2019)	Canada	Cross sectional	Home and community care; long‐term care homes	Health support workers	***Quality of work life*** : Quality of Work Life Measure (13 items, Scale 1–5 (Sirgy et al., 2001)	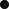				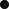	
28	Rai (2013)	The USA	Cross sectional	Nursing homes	Staff	***Quality of working life*** : five subscales chosen from a work‐related quality of life scale (Van Laar et al., 2007)	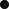	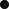	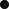		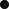	
29	Rai (2015)	The USA	Cross sectional	Long‐term care facilities	Staff	***Quality of working life*** : five subscales chosen from a work‐related quality of life scale (Van Laar et al., 2007)	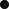	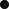	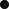		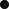	
30	Rosati et al.( 2009)	The USA	Cross sectional	Domiciliary healthcare agency	Staff	The Morehead staff survey	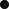	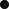			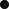	
31	Scanlan & Hazelton (2019)	Australia	Cross sectional	Mental health settings	Occupational therapists	***Job satisfaction***: single‐item: ‘Overall, how satisfied are you with your current job?’ ***Burnout***: Oldenburg Burnout Inventory (OLBI) (E. Demerouti et al., 2010)) ***Professional identity:*** Professional Identity Questionnaire (PIQ) (H. Edwards & Dirette, 2010)			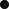			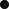
32	Scanlan et al. (2013)	Australia	Cross sectional	Mental health settings	Occupational therapists	***Job satisfaction***: ‘How satisfied are you with your job as a whole?’ ***Burnout***: Oldenburg burnout inventory (OLBI) (Evangelia Demerouti et al., 2003) ***Work engagement***: Utrecht work engagement scale (UWES−17) (W. B. Schaufeli & Bakker, 2004 )			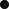			
33	Sebastiano et al. (2017)	Italy	Cross sectional	Nursing homes	Aid nurses; nurses; rehabilitation technicians; people in administration and general services; doctors; social animators; service support; service coordinators; managers; and other professionals	***Positive well‐being***: part of the Job‐Related Affective Well‐being Scale (Warr, 1990)	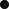		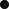			
34	Selamu et al. (2017)	Ethiopia	Qualitative: interviews and focus groups	Primary healthcare facilities	Nurses; health officers; health extension workers	Questions focusing on the concepts of wellbeing, job‐related stress and burnout	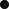	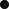	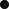			
35	Shier & Graham (2011)	Canada	Qualitative: interviews	Members registered with the Alberta College of Social Workers	Social workers	‘what things at work have the greatest impact on your subjective wellbeing; what things do you do at work to ensure that your wellbeing is provided for; and what aspects of your work life result in high levels of wellbeing and what negatively impacts wellbeing’	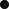	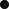				
36	Smith et al. (1994)	The USA	Cross sectional	Domiciliary health agency	Nurses (registered and licensed practice nurses), homemakers, and support staff	***Job satisfaction***: adopted from Hoppock (Hoppock, 1935) and revalidated by McNichols, Stahl and Manley (McNichols et al., 1978) ***Job involvement:*** Lodahl and Keiner (Lodahl & Kejnar, 1965) ***Propensity to remain***: Lyons (Lyons, 1971)	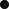		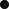			
37	Spoor et al. (2010)	The Netherlands	Protocol paper	Nursing homes	Nursing staff (i.e. nursing assistants, certified nursing assistants, and registered nurses)	***Cognitive, emotional and physical job demands and job resources***: the DISC Questionnaire (DISQ) (van den Tooren & de Jonge, 2008) ***Recovery from work:*** a scale by De Jonge et. al. (De Jonge et al., 2008)		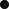	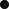	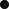	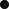	
38	Steinheiser (2018)	The USA	Phenomenological: participants interviewed 3 times	Skilled nursing facility	Skilled nursing facility registered nurses	Questions focusing on ***experiences of caring for older adults***	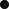	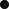	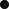	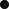		
39	Testad et al. (2010)	Norway	Cross sectional	Dementia care wards at nursing homes	Certified nurse assistant; licensed practical nurse; registered nurse	***Organizational and psychosocial factors*:** General Nordic Questionnaire for Psychosocial and Social Factors at Work (QPSNordic) (Elo et al., 2000)	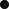	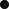	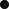	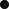		
40	van der Meer et al. (2018)	The Netherlands	Cross sectional	Residential care facility for people with intellectual disabilities	Staff	***The well‐being of professionals*** : the 15‐item Social Production Function Instrument for the Level of Well‐being (Nieboer et al., 2005)			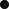	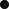		
41	Vermeerbergen et al. (2017)	The selected studies conducted research in: Belgium, the Netherlands, Sweden and the USA	Evidence synthesis	Small‐scale and large‐scale homes	Not applicable	Not applicable	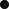	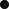	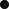			
42	Willemse et al. (2015)	The Netherlands	Cross sectional (LAD‐study)	Large‐scale nursing homes; stand‐alone group living homes in the community	Nursing staff (i.e. (certified) nursing assistants and registered nurses)	***Measures for job characteristics:*** from the Leiden Quality of Work Questionnaire (LQWQ) (van der Doef & Maes, 1999) ***Job related well‐being*** *:* *job satisfaction* ***:*** 3‐item scale from the LQWQ (van der Doef & Maes, 1999) *intent to leave*: 3‐item scale from the LQWQ (van der Doef & Maes, 1999) *emotional exhaustion*: the Dutch version (W. Schaufeli & van Dierendonck, 2000) of the Maslach Burnout Inventory (MBI) (Maslach & Jackson, 1986 ) *personal accomplishment* **:** the 7‐item subscale of the Dutch version of the MBI (Maslach & Jackson, 1986; W. Schaufeli & van Dierendonck, 2000)	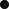	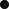	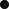			
43	Willemse et al. (2012)	The Netherlands	Cross sectional (LAD‐study)	Nursing homes, homes for the aged with a special care unit for people with dementia, small group living homes	Healthcare workers (i.e. nursing assistants, certified nursing assistants, and registered nurses)	***Measures for job characteristics***: from the Leiden Quality of Work Questionnaire (LQWQ) (van der Doef & Maes, 1999) ***Job satisfaction:*** a 3‐item scale from the LQWQ (van der Doef & Maes, 1999)	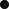	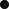	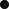			
44	Yoder (2010)	The USA	Mixed method: qualitative was part of the questionnaire	Community hospital	Registered nurses	***Professional quality of life*:** the Professional Quality of Life Scale (ProQOL R‐IV) (Stamm, 2005) ***Qualitative part:*** two questions focusing on compassion fatigue or burnout	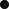	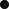	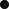	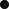	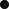	
Studies identified through references												
45	Denton et al. (2002)	Canada	Mixed method: focus groups and questionnaire	Domiciliary care organisations	Visiting home care workers: nurses, therapists, home support workers	Focus groups: health and work life concerns of home care workers Questionnaire ***Intrinsic job satisfaction:*** a list of statements to describe job satisfaction ***Stress***: frequency of experiencing fourteen indicators of stress	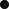	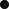	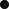	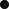		
46	Dutcher & Adams (1994)	The USA	Cross sectional	Home health agencies	Nurses and home health aides	***Work environment*** : Moos's Work Environment scale form Real ^®^ (Moos, 1981)	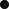	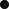		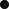		
47	Eustis et al. (1993)	Origin of studies included not reported	Evidence synthesis	Domiciliary care	Care workers providing care for older clients	Not applicable	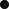	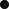				
48	Graham & Shier (2009)	Canada	Qualitative: interviews	Government, non‐profit and private practice	Social work practitioners	Aspects of personal and work life and the profession of social work that have an impact on overall subjective well‐being	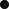	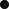			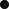	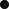
49	Hay (2015)	England	Qualitative: focus groups	Care homes and domiciliary or community care providers	Care workers	Attitudes to low‐paid work, including perspectives on what makes a ‘good’ or a ‘bad’ job, and ideas for improving work	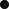	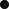				
50	Josefsson et al. (2007)	Sweden	Cross sectional	Municipal elderly care in dementia care and in general care	Registered nurses	Employment conditions, the work situation regarding demands, authority over decisions, and support	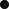	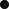				
51	Karlsson et al. (2009)	Sweden	Qualitative: interviews	Residential care homes	Registered Nurses	Experiences as an registered nurse in residential care home	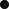	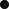	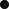			
52	Keesler & Troxel (2020)	The USA	Cross sectional	A not‐for‐profit agency: supporting individuals with intellectual and developmental disabilities and their families	Direct support professionals	***Professional quality of life:*** the Professional Quality of Life (ProQOL) (Stamm, 2010)			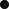			
53	Willemse et al. (2014)	The Netherlands	Cross sectional (LAD‐study)	Group living home care or small‐scale care	Certified nursing assistant, care assistants, registered nurses	***Job characteristics*:** the Leiden Quality of Work Questionnaire (LQWQ) (van der Doef & Maes, 1999)	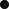	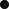				
54	Willemse et al. (2011)	The Netherlands	Mixed method: focus groups and questionnaire (LAD‐study)	Group living home care	Survey: health care staff (i.e. nursing assistants, certified nursing assistants and registered nurses) Focus groups: managers and healthcare professionals, members of the care staff and family members of the residents	***Job satisfaction, intention to leave and the job characteristics*:** the Leiden Quality of Work Questionnaire (van der Doef & Maes, 1999) ***Burnout*:** the Dutch version of the Maslach Burnout Inventory (Maslach & Jackson, 1986 ) the Utrecht Burnout Scale – C (Wilmar Schaufeli & van Dierendonck, 1994; W. Schaufeli & van Dierendonck, 2000) ***Focus groups***: opinion on points of success and improvement of the living arrangements and how care staff, residents, family members, volunteers, management, finances, philosophy of care, policy and environment contribute to this	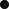	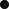	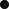			
55	Zoeckler (2018)	The USA	Qualitative: observations and interviews	Domiciliary healthcare agencies	Home health aides; agency leaders	Questions relating to work demand, autonomy on the job, workplace support, and efforts versus reward	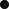	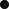	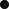	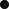		
Studies provided through network of experts												
56	Ricardo Rodrigues (2018)	Austria	Cross sectional (Nordcare)	Domiciliary and residential care	Workers from care homes and domiciliary care	The survey on working conditions (Szebehely, 2017)	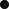	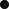	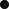	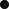		
57	Strandell (2020)	Sweden	Longitudinal (Nordcare)	Disability support and residential and home care	Unionised care workers (home‐helpers, care aides, assistant nurses and similar occupations)	***Workload and time pressure***: Too high workload, and Working short‐staffed ***Problems with autonomy***: Lack of discretion and Distrust from managers ***Lacking support*:** Weak support from supervisor and Weak collegial support ***Relationship to the client*:** Feeling inadequate and Lack of relational reward ***Physical and emotional impact***: Physical and Mental exhaustion	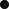	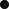	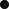	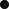		
58	Theobald et al. (2018)	Germany, Japan, Sweden	Cross sectional (Nordcare)	Residential‐ and home care provider	Care workers	the Nordcare survey (Szebehely, 2017 )	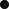	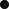	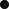	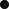		
59	Meagher et al. (2016)	Sweden and Australia	Cross sectional (Nordcare)	Domiciliary‐ and residential care for older and disabled people	Domiciliary care workers	the Nordcare survey (Szebehely, 2017)	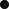	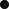	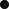	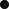		
60	Trydegård (2012)	Denmark, Finland, Norway and Sweden	Cross sectional (Nordcare)	Domiciliary and residential care	Home helpers, assistant nurses, care aides, nurse's aides and practical nurses	***Working conditions:*** the Nordcare survey (Szebehely, 2017)	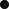	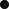	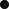	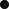		
61	Armstrong et al. (2009)	Canada and the Nordic countries	Mixed method: questionnaires and focus groups	Long‐term residential care	Canada: housekeepers; dietary aides; personal support workers; licensed practical and registered nurses Nordic countries: direct care workers	The Scandinavian data were collected as part of Nordcare. Second source of data is from the comments written on the questionnaires. Third, focus groups were conducted in order to validate the survey results and opportunity to discuss findings and offer additional comments. Fourth, data and research from a variety of sources, including research conducted in Canada and abroad as well as data from a variety of statistical agencies.		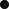		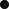	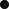	
Studies published between January and September 2021												
62	Aughterson et al. (2021)	The UK	Qualitative: interviews	Hospital, residential, community and primary care settings	A range of frontline professions within health and social care	Topic guide included for example: ‘How would you describe your social life now that social distancing measures have been brought in because of COVID‐19’ ‘In what ways has your work life been impacted by the COVID‐19 pandemic’ How do you feel about the changes that have been brought about by COVID‐19? Have they had impact on your mental health or well‐being?	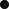	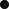	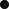		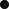	
63	Bensliman et al. (2021)	Belgium	Mixed methods: documentary review, interviews and participatory approach	Domiciliary care	Homecare workers, department directors	The data collection centred around following topics: 1. Historic, process, dynamic of innovation; 2. Innovators profile; 3. Governance system of the organisation; 4. Work conditions and work organisation, workplace wellbeing policy, and workers’ participation; 5. Older adults/caregivers’ profile and nature of relationship with workers; 6. Business model of the organisation	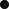	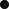	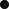			
64	Bielderman et al. (2021)	the Netherlands	Cluster‐randomized controlled trial	Nursing homes	Nursing staff members	**Stress:** the Dutch version of the Maslach Burnout Inventory: the Utrecht Burnout Scale –C’ (UBOS‐C) (Maslach & Jackson, 1986; W. Schaufeli & van Dierendonck, 2000) **Work contentment, job satisfaction and job demands**: two subscales of the Leiden Quality of work Questionnaire for nurses (LQWQ‐ nurses) (S. Maes, 1999), an adaptation of the Leiden Quality of Work Questionnaire (LAKS) (van der Doef & Maes, 1999). **Stress reactions at work**: 2 subscales (emotional reactions at work) of ‘Questionnaire on the Experience and Evaluation of Work’(QEEW) (van Veldhoven & Meijman, 1994)	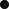	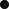	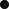			
65	Grasmo et al. (2021)	Norway	Qualitative: interviews	Home care	Home care workers (HCW)	Open ‐ended questions and other additional relevant prompts (e.g., ‘How do you perceive your work conditions?’; ‘What does a typical workday look like for you?’; How does your experience of working conditions influence your health?ʼ; ‘How do your experience work conditions influence your health?’; ‘Can you describe how you cope with challenges in your workday and how it affects your health?’).	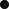	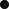	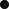	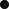		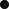
66 and 67	McFadden, Gillen, et al. (2021) and McFadden, Ross, et al. 2021)	the UK	Cross sectional	Health and social care (only data focusing on social care are extracted	Professionals working in social care and work	**Work‐related quality of life:** The Work‐Related Quality of Life (WRQOL) scale (Van Laar et al., 2007): control at work, general wellbeing, home–work interface, job career satisfaction, stress at work, and working conditions.	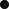	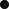	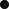		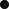	
68	Sebastiano et al. (2021	Italy	Cross sectional	Nursing home	nurses, nurse‐aids, physiotherapists, recreational therapists, professional educators and doctors;	**The well‐being of caregivers**: the Job‐Related Affective Well‐being Scale. (P. G. Warr, 1990)	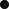	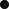	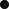			

Abbreviations: JC, job characteristics; MWaH, mental wellbeing and health; OC, organisational characteristics; PI, professional Identity; PWaH, physical wellbeing and health; S, spill‐over from work to home.

### 3.3 Definitions of WRQoL

Based on the 68 publications included in this study, there is an absence of an agreement on a definition of WRQoL. Furthermore, researchers used a variety of terms to describe this concept including: work psychosocial characteristics (Iecovich, 2011), job characteristics (Willemse et al., 2012), work environment (Dutcher & Adams, 1994), individuals' perceptions of the impact of their work (Denton et al., 2002), working conditions (Eustis et al., 1993a; Strandell, 2020), work situation (Josefsson et al., 2007), work‐related experiences (Karlsson et al., 2009; Zoeckler, 2018), the factors that comprised a good or a bad job (Hay, 2015), work‐related problems (Strandell, 2020), work situation (Theobald et al., 2018) and the care workers' assessment of their working conditions from the perspective of their own health and wellbeing (Trydegård, 2012). Very few studies provided an explicit definition of WRQoL (Arts et al., 2001; Brousseau et al., 2008; Durkin et al., 2016; Itzick et al., 2018; Malherbe & Hendriks, 2004; Perreira et al., 2019; Rai, 2015; Scanlan et al., 2013; Sebastiano et al., 2021; Vermeerbergen et al., 2017) and of those even fewer linked those definitions to specific theoretical models (Arts et al., 2001; Brousseau et al., 2008; Perreira et al., 2019; Sebastiano et al., 2021; Vermeerbergen et al., 2017). Examples of definitions include: p13: "quality of working life is not a distinct concept, but can be associated with aspects such as job satisfaction, job involvement, motivation, productivity, health, safety, and wellbeing" (Arts et al., 2001); p110 and p328 respectively: "professional quality of life consists of compassion, satisfaction, burnout, and compassion fatigue" (Durkin et al., 2016; Itzick et al., 2018), and p35: "quality of work life is a management philosophy that enhances personnel's dignity through the creation of favourable working conditions, initiating changes in the culture of the organisation, improving the physical and emotional wellbeing of employees and creating opportunities for growth and development in an environment that promotes job satisfaction” (Malherbe & Hendriks, 2014).

### 3.4 Key components of WRQoL

We conducted a thematic analysis of definitions of WRQoL, measures, and factors associated with WRQoL or general wellbeing and identified six key components: organisational characteristics; job characteristics; mental wellbeing and health; physical wellbeing and health; spill‐over from work to home; and professional identity. Each key component consists of several sub‐themes. For example, job characteristics include job–person match; autonomy/control at work; time; responsibility for people; learning and growth opportunities/self‐actualisation; meaningful work and feedback from work. The full list of key components, description and examples of corresponding items are presented in Table 2. Table 1 shows each study and its corresponding key component of WRQoL and Table S3 includes a full list of items that were coded in the thematic analysis (some items were assigned to more than one component of WRQoL).

**TABLE 2:** Description of key areas of work‐related quality of life (WRQoL) and examples of items they reflect

Key components of WRQoL	Sub‐themes	Description	Examples of items
Organisational characteristics	Working Culture	Norms of behaviour, thinking and emotional intelligence, values and basic assumptions, beliefs, routines, traditions, sense‐making perspectives shared by members of an organisation including aspects that make it easy/difficult to adjust to the work (working conditions).	Working environment; Role clarity; Communication; Working hours; Job security; Rules and procedures; Paperwork and bureaucracy; Pay and other benefits; Sufficient human and material resources; Diversity and Equality
	Working Climate	Members’ perception of working culture. Feeling or atmosphere people get in the organisation on either a day‐to‐day basis or just generally.	Leadership style; Encouragement of new ideas; Recognition; Social support/relationships/trust; Influence/Participation/Decentralisation; Commitment to quality of services; Positive experiences at work; Job satisfaction;
Job characteristics	Job–person match	The ability/personal resources to do the job. Matching the right person to the job. Matching individual's cognitive abilities, interests, and personality resources to those required for success in a particular job.	Adaptability; Empathy and altruism; Self‐efficacy; Cognitive/emotional/physical demands and resources; Encounters with death
	Autonomy/Control at work	The degree to which a job provides substantial freedom, independence, and discretion to the individual in scheduling the work and determining the tasks.	Freedom in decision‐making; Allowing the professional to take new approaches to care based on their knowledge; Flexibility; Managing one's own work; Resolution in problem‐solving; Being able to prioritise care activities
	Time	Having the time required to do the job.	Tasks taking up too much time and detracting from the more important functions of the job; Spending time with and caring for the residents; Time pressure; Too many things to juggle during the work day; Time for appropriately performing nursing and care activities, and occupational requirements
	Responsibility for people	Making decisions and caring for others.	Fear of ‘getting it wrong’; Complex needs; Increasing violence, abuse & behavioural problems; Connection with clients
	Learning and growth opportunities/self‐actualisation	Providing individuals with tools and skills to perform their role; fulfilment of one's potential.	Development; Growth in the organisation
	Meaningful work	The degree to which a job has a substantial impact on the lives or work of other people.	Getting a lot out of working with the clients
	Feedback from work	The degree to which carrying out the work activities required by the job results in the employee obtaining information about the effectiveness of their performance. The feeling of doing a good/bad job and being appreciated (or not) for their work.	Feelings of value and self‐esteem; Feeling inadequate; Personal accomplishment
Mental wellbeing and health	Compassion satisfaction	The positive feeling associated with knowing that the professional has in some way helped another.	Subjective experience of happiness
	Compassion fatigue	Occurs as a result of hearing about a traumatizing event that a person has experienced.	Feeling emotionally and physically exhausted
	Burnout/Work engagement	Work engagement is often considered the theoretical opposite of burnout: while burnout is defined in negative terms, work engagement is seen when employees are motivated, enthusiastic and energised by their work. Work engagement can be seen as ‘health at work’ rather than simply the absence of ‘sickness at work’ (burnout).	Feeling irritable or frustrated while working; Finding work overwhelming
	Mental well‐being	Thoughts and feelings and how a person copes with them.	General Well‐Being; Life satisfaction; Subjective experience of happiness
Physical wellbeing and health		Being able to engage in different activities (physical, social etc.) without e.g. experiencing pain.	Physical injury at work; Time of work due to the work‐related injury/injuries
Spill‐over from work to home		‘Taking’ work home including work‐life balance.	Work‐related thoughts when off duty
Professional Identity		Perception of ourselves within our occupational context and how we communicate this to others.	Care workers see themselves as a professional; Employer(s) respect care workers as a professional

Abbreviations: WRQoL, work‐related quality of life.

### 3.5 Measures of WRQoL

The overview of measures of WRQoL is included in Table 1 (Column: Measures of WRQoL). Most of the studies used a combination of different questionnaires to measure different components of WRQoL and none covered all six components of WRQoL as identified in this scoping review. The most common components of WRQoL measured in the studies were: organisational characteristics, job characteristics and mental wellbeing and health. Questionnaires that explicitly measure WRQoL in social care or community health care context are: Quality of Work Life Measure (Sirgy et al., 2001), the Professional Quality of Life (ProQOL) Scale (Stamm, 2009) and its revised version, the Professional Quality of Life Scale‐Revised (ProQOL) (Stamm, 2005)); Leiden Quality of Work Questionnaire (LQWQ) (van der Doef & Maes, 1999) and its adaptation (Maes et al., 1999)**;** the Nordcare survey (Szebehely et al., 2017); the 15‐item version of the Social Production Function Instrument for the Level of Well‐being (Nieboer et al., 2005); the Work‐related quality of life scale (Van Laar et al., 2007); and the Job‐Related Affective Well‐being Scale (Warr, 1990). While some measures do not explicitly measure WRQoL, the areas covered overlap with those measured by the WRQoL instruments. Examples of such questionnaires/concepts include: work psychosocial characteristics measured by a 30 item questionnaire (Pikhart et al., 2001); and work environment measured by Moos's Work Environment scale form Real ^®^ (Moos, 1981).

### 3.6 Strategies implemented and evaluated in adult social care that addressed social care staff's WRQoL

We identified only two randomised controlled trials that measured social care staff's WRQoL (Berendonk et al., 2019; Bielderman et al., 2021). The first one (Berendonk et al., 2019) tested a nursing intervention (DEMIAN) in 20 German long‐term care facilities and its effects on frontline staff’ job satisfaction, motivation and work strain. The DEMIAN intervention provided tools for staff to improve situational wellbeing and experiences of meaning and purpose for people living with dementia. This in turn led to significantly decreased time pressure and decreased job dissatisfaction for frontline staff allocated to intervention group. The second one (Bielderman et al., 2021) tested the short‐term (3 months) and long‐term (9 months) effects of the TENSE training program on experienced stress, work contentment, and stress reactions at work in nursing staff working in 18 Dutch dementia special care units. The authors reported that the TENSE training program had no effect on any of the components related to social care staff's WRQoL.

Based on the qualitative and observation studies included in the review, several suggestions about how to improve different components of WRQoL have been identified. For example, participants from two UK Social Service Departments suggested following: to employ more staff, especially trained, permanent, competent staff and to replace staff who leave as quickly as possible. This was followed by more support and understanding of working conditions and the nature of the job – recognition and appreciation of hard work. Staff also wanted more training, both internal and external, including management training, especially before new procedures are implemented (Coffey et al., 2009). Another study (Kemper et al., 2008) conducted among staff working at US skilled nursing facilities, assisted living facilities, and home care agencies recommended the following to improve their job: increased compensation, improved work‐relationships, improved staffing, and improved management systems. Next, care workers from private, for‐profit care homes and domiciliary (or community) care providers in England (Hay, 2015) wanted: initiatives that would offer workers a greater level of security in their lives outside work (e.g. paying the Living Wage, assistance with childcare, paid sick leave etc.); initiatives intended to make care workers feel better supported at work (e.g. more time at work, better work‐related training, more involvement in decision‐making and a more supportive working environment) and lastly initiatives aimed at enhancing workers’ financial security such as pensions, financial assistance and financial advice. Lastly, a study conducted in Belgium among homecare workers and departmental directors gave similar suggestions: to set up more participation and specific meetings to discuss well‐being at work, work organisation adjustment, and sense of innovation; humanisation of work relations; having more social support, listening, trust, and recognition from the hierarchy and management (Bensliman et al., 2021).

## 4 DISCUSSION

The aim of this scoping review was to understand what the existing definitions of WRQoL in adult social care are, its main components and what measures were used to assess it. While there is a substantial research relevant to WRQoL, there is an absence of agreement on the definition of WRQoL in the context of adult social care with different terms to describe WRQoL. Only seven studies provided explicit definitions of WRQoL and of those, only five linked their definition to specific theoretical models (e.g. job demand‐control model (Karasek, 1979), job characteristics model (Hackman & Oldham, 1976), salutogenetic model of health (Antonovsky, 1996), Watson's philosophy of human caring (Watson, 2006), and theory of reasoned action (Martin, 1980)). However, based on the thematic analysis of the evidence gathered for this study we conclude that WRQoL in adult social care is a complex concept including six key components: organisational characteristics, job characteristics, mental wellbeing and health, physical wellbeing and health, spill‐over from work to home, and professional identity. As there is no agreement on what WRQoL is, it is not surprising that the majority of the studies used different tools to measure different components of WRQoL. Lastly, studies of strategies implemented and evaluated in adult social care to address WRQoL are almost entirely lacking. This scoping review builds on existing evidence syntheses in adult social care (André et al., 2014; Arts et al., 2001; Vermeerbergen et al., 2017), however, it is considerably more robust than previous studies as it combined several key strengths including: breadth of the context and participants included in the present review (all aspects of adult social care e.g. home care, residential care, live‐in care etc. and job roles were included); breadth of the literature searches including grey literature, references from identified studies and contacting network of experts on the topic; systematic approach to literature search, data extraction and data synthesis following accepted best practice with independent screening of full‐text articles for inclusion (Peters et al., 2017; Popay et al., 2006; Tricco et al., 2018).

The first study that measured WRQoL in social care was published in 1985 (MacEachron et al., 1985) and almost 36 years and 67 studies later there is still no agreement on what WRQoL is in the context of adult social care. This is not surprising, as previous evidence syntheses either in social care (André et al., 2014; Arts et al., 2001; Vermeerbergen et al., 2017) or in other professions, e.g. health care settings (Campbell et al., 2019; Nowrouzi et al., 2016) came to similar conclusions. There are four key factors raised in the literature explaining the lack of agreed definition: 1. different understanding of WRQoL across different disciplines (e.g. economics, medicine (Vendrig & Schaafsma, 2018), social sciences (Cummins, 2005; Cummins et al., 2004)); 2. interchangeable use of terms describing the concept of WRQoL (e.g. work psychosocial characteristics (Iecovich, 2011), job characteristics (Willemse et al., 2012), work environment (Dutcher & Adams, 1994) etc.); 3. lack of empirical testing of proposed models (Cummins, 2005); and 4. the need for new models of WRQoL that reflect changes in the workplace (Loscocco & Roschelle, 1991). Furthermore, adult social care itself has changed over time, the content of work and job role varies in different context (e.g. home care, residential care etc.) and differs between countries. This raises the question of whether ‘one definition fits all’ is the best way to take research in this area forward. However, lack of agreement on what WRQoL is in adult social care, creates several challenges, which are likely to hinder efforts to enhance the experience of workers in this sector. These challenges range from the selection of measures, to the ability to conduct comparative studies and more importantly in the development of interventions and evaluations informing policymakers and employers on how to improve staff's WRQoL. One approach to overcome some of these challenges is to establish an agreed definition of WRQoL. Researchers should then be explicit about which components they intend to measure and why. This scoping review makes steps towards such agreement as we have identified and defined six key components of WRQoL in adult social care. However, it is important to test whether these components specifically speak to the experiences of social care workers as none of the included studies used models, definitions, or tools that were specifically developed in the context of adult social care.

Most of the studies included in this scoping review, rather than measuring WRQoL as a specific concept, used a package of tools to measure different components of WRQoL. For example, Scanlan et al., 2013 (Scanlan et al., 2013) measured job satisfaction, burnout and work engagement with different questionnaires. While this allows a flexible approach, e.g. measuring only the components of WRQoL of interest, such approaches may be quite time consuming for people filling in questionnaires and create difficulties comparing different studies. Given the time pressures in adult social care, lengthy questionnaires may lead to decreased engagement with such tools among care staff. One brief questionnaire measuring the key components of WRQoL may be a better alternative. In this scoping review, we have identified seven measures of WRQoL: Quality of Work Life Measure (Sirgy et al., 2001), the Professional Quality of Life (ProQOL) Scale (Stamm, 2009) and its revised version, the Professional Quality of Life Scale‐Revised (ProQOL) (Stamm, 2005)); Leiden Quality of Work Questionnaire (LQWQ) (van der Doef & Maes, 1999) and its adaptation (Maes et al., 1999)**;** the Nordcare survey (Szebehely et al., 2017); the 15‐item version of the Social Production Function Instrument for the Level of Well‐being (Nieboer et al., 2005); the Work‐related quality of life scale (Van Laar et al., 2007) and the Job‐Related Affective Well‐being Scale (Warr, 1990). Interestingly, the Professional Quality of Life (ProQOL) Scale (Stamm, 2009) and its revised version (Stamm, 2005) was the most often used measure by different research teams (four) (Dunkin et al., 1996; Itzick et al., 2018; Keesler & Troxel, 2020; Yoder, 2010). The only other measure used by different teams was the Work‐related quality of life scale (Van Laar et al., 2007) used by (Rai, 2013, 2015) and more recently used in a study focusing on health and WRQoL and coping among health and social care staff while working during the COVID‐19 pandemic in the UK (McFadden, Gillen, et al., 2021). Before new measures are developed, we recommend a systematic review of the psychosocial properties of the identified measures of WRQoL. Such review would help to understand whether these measures are valid and reliable measures of WRQoL and whether they are feasible to be used in adult social care either in their existing forms or with some adaptations.

One of the key research questions of this scoping review was to identify strategies implemented and evaluated in adult social care that addressed social care staff's WRQoL. We identified only two randomised controlled trials (Berendonk et al., 2019; Bielderman et al., 2021) addressing this question. While in the studies included in this scoping review different suggestions were raised by participants on how different components of WRQoL may be improved, these have not been further empirically tested. This indicates a considerable gap in the present evidence.

Finally, the results of this study have to be interpreted in light of its limitations. Firstly, while we screened almost 8000 articles from variety of resources, given the variety of terms used to describe WRQoL it is possible that there are additional studies of relevance that we did not include. Given the number of articles screened and the breadth of literature resources, it is however unlikely that these would change our findings. Secondly, the social care sector has changed over time and varies between different countries, including terminology and work content. This made it a challenging task to determine the exact nature of the job of participants of included studies and to ensure that all relevant studies were correctly identified. Again, however, our systematic and broad approach to identifying literature minimised this risk. Lastly, we only included studies written in English, potentially excluding relevant non‐English evidence.

## 5 CONCLUSION

The scoping review itself highlights several key messages for researchers. Firstly, researchers should provide explicit definition of WRQoL and/or its components with the justification for its choice– either to link it to an existing concept or to an existing empirical evidence. If it is clear that they measure the same concept/component, researchers could compare the results from different studies. Secondly, while we cannot recommend a specific tool to measure WRQoL in adult social care, this scoping review provides a catalogue of existing tools measuring different components of WRQoL in different contexts of adult social care. Lastly, given the inconsistency in definition of WRQoL and its measurement, and lack of strategies aiming to improve WRQoL and/or its components, it is not possible to provide robust recommendations to policy makers or national organisations on how to improve WRQoL among their staff.

## 6 CONSENT FOR PUBLICATION

Not applicable.

## CONFLICT OF INTEREST

The authors have no relevant financial or non‐financial interests to disclose.

## AUTHORS' CONTRIBUTIONS

The initial idea for a scoping review on the topic came from SP, A‐M T and SH. The core sections of the review were formulated by BS, NB, SP, A‐M T and SH. The search strategy was devised by BS and Abigail Heath (AH). Screening records was undertaken by BS, NB, and SP, and any discrepancies were resolved through discussion with A‐M T and SH. Data from these records were extracted by BS who also undertook the qualitative thematic analysis, with NB and SP examining the qualitative coding and naming of thematic domains for consistency. BS designed all figures and tables, with comments and suggestions from NB and SP. Preparation of the manuscript was undertaken by BS, with considerable input from all authors.

## ETHICS APPROVAL

This work did not involve human participants or animals and so no ethical approval was required.

## Supporting information

Table S1

Table S2

Table S3

## Data Availability

Available from the corresponding author: Barbora Silarova, PhD, Personal Social Services Research Unit, University of Kent, Cornwallis Central, Canterbury, CT2 7NF, UK, email: B.Silarova@kent.ac.uk.
