# Supplementary material for: Understanding and measuring the work‐related quality of life among those working in adult social care: A scoping review
Source: Health Soc Care Community. 2022 Jan 23;30(5):1637–64. doi: 10.1111/hsc.13718 (PMC9543435; doi:10.1111/hsc.13718)
Supplement: Supplementary file 1 — Table S1 [file HSC-30-1637-s001.docx]

**Article title:** Understanding and measuring the work-related quality of life among those working in adult social care: A scoping review

**Journal name:** Health and Social Care in the Community

**Author names:** Barbora Silarova, Nadia Brookes, Sinead Palmer, Ann-Marie Towers, Shereen Hussein

**Affiliation and e-mail address of the corresponding author**: Barbora Silarova, PhD, Personal Social Services Research Unit, University of Kent, Cornwallis Central, Canterbury, CT2 7NF, UK

Email: B.Silarova@kent.ac.uk

**Online Resource 1** Table 1. Eligibility criteria

|  | **Inclusion** | **Exclusion** |
| --- | --- | --- |
| **Type of Participants** | Direct care workers.  People working in managerial and supervisory roles in the adult social care sector.  Registered professionals working in adult social care.  Nursing aides, nursing assistants and nursing health care staff, health care assistants, and other allied health care professions working in community health settings. | Social care workers and registered professionals in children’s services, hospitals, hospices or education.  Informal carers (e.g. family friends or neighbours). |
| **Concept** | Job-related well-being  Job-related quality of life  Quality of Work Life  Quality of Working Life  Work Life Quality  Work-related quality of life  Work-related wellbeing  We also included studies focusing on general well-being or quality of life if:   - general well-being or quality of life were measured as dependent variables AND - conducted among those working in adult social care AND - the independent variable was a measure of any aspect of the social care work (e.g. salary, stress, job satisfaction etc.). | Work-related quality of life defined only through one dimension, e.g.:  Perceived stress  Job stress  Burnout  Compassion fatigue  Job satisfaction  Job demands  Job resources etc.  We also excluded studies focusing on general well-being or general quality of life if they:   - did not include individuals working in adult social care OR - general well-being or quality of life were measured as independent variables OR - independent predictors are not measures of any aspects of social care job/work (e.g. gender, age) |
| **Context** | Adult social care  Community health setting | Organisations that do not provide adult social or community health care. |
| **Type of study** | Primary research studies: qualitative, quantitative or mixed methods. Systematic reviews, meta-analyses and other types of evidence synthesis.  Protocols. | Abstracts, letters, editorial, commentaries, discussion and opinion-based papers, books and chapters in books. |
| **Language:** | English | Any other language |
| **Time restriction** | None | NA |
