# Supplementary material for: Understanding and measuring the work‐related quality of life among those working in adult social care: A scoping review
Source: Health Soc Care Community. 2022 Jan 23;30(5):1637–64. doi: 10.1111/hsc.13718 (PMC9543435; doi:10.1111/hsc.13718)
Supplement: Supplementary file 2 — Table S2 [file HSC-30-1637-s002.docx]

**Article title:** Understanding and measuring the work-related quality of life among those working in adult social care: A scoping review

**Journal name:** Health and Social Care in the Community

**Author names:** Barbora Silarova, Nadia Brookes, Sinead Palmer, Ann-Marie Towers, Shereen Hussein

**Affiliation and e-mail address of the corresponding author**: Barbora Silarova, PhD, Personal Social Services Research Unit, University of Kent, Cornwallis Central, Canterbury, CT2 7NF, UK

Email: B.Silarova@kent.ac.uk

**Online Resource 2** Full search strategy

Table 1. PubMed search strategy

|  | **Search Term Category** | **Query** | **Search details: Mesh + key word** |
| --- | --- | --- | --- |
| **1** | **Work-related quality of life** | Work-related wellbeing | Work-related[All Fields] AND wellbeing[All Fields] |
| **2** |  | Work-related quality of life | Work-related[All Fields] AND ("quality of life"[MeSH Terms] OR ("quality"[All Fields] AND "life"[All Fields]) OR "quality of life"[All Fields]) |
| **3** |  | "Work life quality" | "Work life quality"[All Fields] |
| **4** |  | "Quality of work* life" | "Quality of work* life"[All Fields] |
| **5** |  | job-related well-being | job-related[All Fields] AND ("health"[MeSH Terms] OR "health"[All Fields] OR "well"[All Fields] OR "well being"[All Fields]) |
| **6** |  | Job related quality of life | Job[All Fields] AND related[All Fields] AND ("quality of life"[MeSH Terms] OR ("quality"[All Fields] AND "life"[All Fields]) OR "quality of life"[All Fields]) |
| **7** |  | 1 OR 2 OR 3 OR 4 OR 5 OR 6 | (((((Job[All Fields] AND related[All Fields] AND ("quality of life"[MeSH Terms] OR ("quality"[All Fields] AND "life"[All Fields]) OR "quality of life"[All Fields])) OR (job-related[All Fields] AND ("health"[MeSH Terms] OR "health"[All Fields] OR "well"[All Fields] OR "well being"[All Fields]))) OR "Quality of work* life"[All Fields]) OR "Work life quality"[All Fields]) OR (Search[All Fields] AND Work-related[All Fields] AND ("quality of life"[MeSH Terms] OR ("quality"[All Fields] AND "life"[All Fields]) OR "quality of life"[All Fields]))) OR (Work-related[All Fields] AND wellbeing[All Fields]) |
| **8** | **People working in adult social care or community health settings** | Worker | Worker[All Fields] |
| **9** |  | "Support worker" | "Support worker"[All Fields] |
| **10** |  | Staff | Staff[All Fields] |
| **11** |  | Registered manager | Registered[All Fields] AND manager[All Fields] |
| **12** |  | "Personal assistant" | "Personal assistant"[All Fields] |
| **13** |  | "Personal support workers" | "Personal support workers"[All Fields] |
| **14** |  | Nursing health care staff | ("Nurs Health Care"[Journal] OR "Nurs Health Care (Winfield)"[Journal] OR "Int Arch Nurs Health Care"[Journal] OR ("nursing"[All Fields] AND "health"[All Fields] AND "care"[All Fields]) OR "nursing health care"[All Fields]) AND staff[All Fields] |
| **15** |  | "Nursing assistant" | "Nursing assistant"[All Fields] |
| **16** |  | "Nursing aide" | "Nursing aide"[All Fields] |
| **17** |  | Nurse | "nurses"[MeSH Terms] OR "nurses"[All Fields] OR "nurse"[All Fields] |
| **18** |  | "Home care workers" | "Home care workers"[All Fields] |
| **19** |  | "Home health care aides" | "Home health care aides"[All Fields] |
| **20** |  | "Home care attendants" | "Home care attendants"[All Fields] |
| **21** |  | "Home helper" | "Home helper"[All Fields] |
| **22** |  | "Home aide" | "Home aide"[All Fields] |
| **23** |  | "Health support workers" | "Health support workers"[All Fields] |
| **24** |  | "Health care workers" | "Health care workers"[All Fields] |
| **25** |  | "Health care assistants" | "Health care assistants"[All Fields] |
| **26** |  | Employee | Employee[All Fields] |
| **27** |  | "Care worker" | "Care worker"[All Fields] |
| **28** |  | "Care staff" | "Care staff"[All Fields] |
| **29** |  | "Care assistant" | "Care assistant"[All Fields] |
| **30** |  | Carer | "Care assistant"[All Fields] |
| **31** |  | "Care aide" | "Care aide"[All Fields] |
| **32** |  | "Physician assistant" | "Physician assistant"[All Fields] |
| **33** |  | 8 OR 9 OR 10 OR 11 OR 12 OR 13 OR 14 OR 15 OR 16 OR 17 OR 18 OR 19 OR 20 OR 21 OR 22 OR 23 OR 24 OR 25 OR 26 OR 27 OR 28 OR 29 OR 30 OR 31 OR 32 | (((((((((((((((((((((((Worker[All Fields] OR "Support worker"[All Fields]) OR Staff[All Fields]) OR (Registered[All Fields] AND manager[All Fields])) OR "Personal assistant"[All Fields]) OR "Personal support workers"[All Fields]) OR (("Nurs Health Care"[Journal] OR "Nurs Health Care (Winfield)"[Journal] OR "Int Arch Nurs Health Care"[Journal] OR ("nursing"[All Fields] AND "health"[All Fields] AND "care"[All Fields]) OR "nursing health care"[All Fields]) AND staff[All Fields])) OR "Nursing assistant"[All Fields]) OR "Nursing aide"[All Fields]) OR ("nurses"[MeSH Terms] OR "nurses"[All Fields] OR "nurse"[All Fields])) OR "Home care workers"[All Fields]) OR "Home health care aides"[All Fields]) OR "Home care attendants"[All Fields]) OR "Home helper"[All Fields]) OR "Home aide"[All Fields]) OR "Health support workers"[All Fields]) OR "Health care workers"[All Fields]) OR "Health care assistants"[All Fields]) OR Employee[All Fields]) OR "Care worker"[All Fields]) OR "Care staff"[All Fields]) OR "Care assistant"[All Fields]) OR ("caregivers"[MeSH Terms] OR "caregivers"[All Fields] OR "carer"[All Fields])) OR "Care aide"[All Fields]) OR "Physician assistant"[All Fields] |
| **34** | To map the concept of work-related quality of life among people working in adult social care or community health settings. | 7 AND 33 | ((((((((((((((((((((((((Worker[All Fields] OR "Support worker"[All Fields]) OR Staff[All Fields]) OR (Registered[All Fields] AND manager[All Fields])) OR "Personal assistant"[All Fields]) OR "Personal support workers"[All Fields]) OR (("Nurs Health Care"[Journal] OR "Nurs Health Care (Winfield)"[Journal] OR "Int Arch Nurs Health Care"[Journal] OR ("nursing"[All Fields] AND "health"[All Fields] AND "care"[All Fields]) OR "nursing health care"[All Fields]) AND staff[All Fields])) OR "Nursing assistant"[All Fields]) OR "Nursing aide"[All Fields]) OR ("nurses"[MeSH Terms] OR "nurses"[All Fields] OR "nurse"[All Fields])) OR "Home care workers"[All Fields]) OR "Home health care aides"[All Fields]) OR "Home care attendants"[All Fields]) OR "Home helper"[All Fields]) OR "Home aide"[All Fields]) OR "Health support workers"[All Fields]) OR "Health care workers"[All Fields]) OR "Health care assistants"[All Fields]) OR Employee[All Fields]) OR "Care worker"[All Fields]) OR "Care staff"[All Fields]) OR "Care assistant"[All Fields]) OR ("caregivers"[MeSH Terms] OR "caregivers"[All Fields] OR "carer"[All Fields])) OR "Care aide"[All Fields]) OR "Physician assistant"[All Fields]) AND ((((((Job[All Fields] AND related[All Fields] AND ("quality of life"[MeSH Terms] OR ("quality"[All Fields] AND "life"[All Fields]) OR "quality of life"[All Fields])) OR (job-related[All Fields] AND ("health"[MeSH Terms] OR "health"[All Fields] OR "well"[All Fields] OR "well being"[All Fields]))) OR "Quality of work* life"[All Fields]) OR "Work life quality"[All Fields]) OR (Search[All Fields] AND Work-related[All Fields] AND ("quality of life"[MeSH Terms] OR ("quality"[All Fields] AND "life"[All Fields]) OR "quality of life"[All Fields]))) OR (Work-related[All Fields] AND wellbeing[All Fields])) |

Table 2. CINAHL, Abstracts in Social Gerontology, APA PsycInfo search strategy

|  | **Search Term Category** | **Search modes - Boolean/Phrase** |
| --- | --- | --- |
| **1** | **Work-related quality of life** | TX Work-related wellbeing |
| **2** |  | TX Work-related quality of life |
| **3** |  | TX "Work life quality" |
| **4** |  | TX "Quality of work* life" |
| **5** |  | TX job-related well-being |
| **6** |  | TX Job related quality of life |
| **7** |  | 1 OR 2 OR 3 OR 4 OR 5 OR 6 |
| **8** | **People working in adult social care or community health settings** | TX Worker |
| **9** |  | TX "Support worker" |
| **10** |  | TX Staff |
| **11** |  | TX Registered manager |
| **12** |  | TX "Personal assistant" |
| **13** |  | TX "Personal support workers" |
| **14** |  | TX Nursing health care staff |
| **15** |  | TX "Nursing assistant" |
| **16** |  | TX "Nursing aide" |
| **17** |  | TX Nurse |
| **18** |  | TX "Home care workers" |
| **19** |  | TX "Home health care aides" |
| **20** |  | TX "Home care attendants" |
| **21** |  | TX "Home helper" |
| **22** |  | TX "Home aide" |
| **23** |  | TX "Health support workers" |
| **24** |  | TX "Health care workers" |
| **25** |  | TX "Health care assistants" |
| **26** |  | TX Employee |
| **27** |  | TX "Care worker" |
| **28** |  | TX "Care staff" |
| **29** |  | TX "Care assistant" |
| **30** |  | TX Carer |
| **31** |  | TX "Care aide" |
| **32** |  | TX "Physician assistant" |
| **33** |  | 8 OR 9 OR 10 OR 11 OR 12 OR 13 OR 14 OR 15 OR 16 OR 17 OR 18 OR 19 OR 20 OR 21 OR 22 OR 23 OR 24 OR 25 OR 26 OR 27 OR 28 OR 29 OR 30 OR 31 OR 32 |
| **34** | To map the concept of work-related quality of life among people working in adult social care or community health settings. | 7 AND 33 |

NOTE: Database CINAHL Plus with Full Text, Interface - EBSCOhost Research Databases, Search Screen - Advanced Search, Expanders - Apply equivalent subjects, Search modes - Boolean/Phrase

Note: Database Abstracts in Social Gerontology, Interface EBSCOhost Research Databases, Search Screen - Advanced Search, Expanders - Apply equivalent subjects, Search modes - Boolean/Phrase.

Note: Database - APA PsycInfo, Interface - EBSCOhost Research Databases, Search Screen - Advanced Search, Expanders - Apply equivalent subjects, Search modes - Boolean/Phrase

Social Care Online database search strategy

| concept   [ -  AllFields:'Work-related wellbeing'   - OR AllFields:'Work-related quality of life'   - OR AllFields:'"Work life quality"'   - OR AllFields:'"Quality of work* life"'   - OR AllFields:'job-related well-being'   - OR AllFields:'Job related quality of life' ]  **AND**  participants   [ -  AllFields:'"Physician assistant"'   - OR AllFields:'Worker'   - OR AllFields:'"Support worker"'   - OR AllFields:'Staff'   - OR AllFields:'Registered manager'   - OR AllFields:'"Personal assistant"'   - OR AllFields:'"Personal support workers"'   - OR AllFields:'Nursing health care staff'   - OR AllFields:'"Nursing assistant"'   - OR AllFields:'"Nursing aide"'   - OR AllFields:'Nurse'   - OR AllFields:'"Home care workers"'   - OR AllFields:'"Home health care aides"'   - OR AllFields:'"Home care attendants"'   - OR AllFields:'"Home helper"'   - OR AllFields:'"Home aide"'   - OR AllFields:'"Health support workers"'   - OR AllFields:'"Health care workers"'   - OR AllFields:'"Health care assistants"'   - OR AllFields:'Employee'   - OR AllFields:'"Care worker"'   - OR AllFields:'"Care staff"'   - OR AllFields:'"Care assistant"'   - OR AllFields:'Carer'   - OR AllFields:'"Care aide"']  Cochrane Review database search strategy  #1 (Work-related wellbeing)  #2 (Work-related quality of life)  #3 ("Work life quality")  #4 ("Quality of working life")  #5 (job-related well-being)  #6 (Job related quality of life)  #7 (#1 OR #2 OR #3 OR #4 OR #5 OR #6)  #8 (Worker)  #9 ("Support worker")  #10 (Staff)  #11 (Registered manager)  #12 ("Personal assistant")  #13 ("Personal support workers")  #14 (Nursing health care staff)  #15 ("Nursing assistant")  #16 ("Nursing aide")  #17 (Nurse)  #18 ("Home care workers")  #19 ("Home health care aides")  #20 ("Home care attendants")  #21 ("Home helper")  #22 ("Home aide")  #23 ("Health support workers")  #24 ("Health care workers")  #25 ("Health care assistants")  #26 (Employee)  #27 ("Care worker")  #28 ("Care staff")  #29 ("Care assistant")  #30 (Carer)  #31 ("Care aide")  #32 ("Physician assistant")  #33 ((8-#32))  #34 (#7 AND #33) |
| --- |

Social Policy and Practice database search strategy

((Work-related wellbeing or Work-related quality of life or "Work life quality" or "Quality of work* life" or job-related well-being or Job related quality of life) and (Worker or "Support worker" or Staff or Registered manager or "Personal assistant" or "Personal support workers" or Nursing health care staff or "Nursing assistant" or "Nursing aide" or Nurse or "Home care workers" or "Home health care aides" or "Home care attendants" or "Home helper" or "Home aide" or "Health support workers" or "Health care workers" or "Health care assistants" or Employee or "Care worker" or "Care staff" or "Care assistant" or Carer or "Care aide" or "Physician assistant")).af.
