# Supplementary material for: Understanding and measuring the work‐related quality of life among those working in adult social care: A scoping review
Source: Health Soc Care Community. 2022 Jan 23;30(5):1637–64. doi: 10.1111/hsc.13718 (PMC9543435; doi:10.1111/hsc.13718)
Supplement: Supplementary file 3 — Table S3 [file HSC-30-1637-s003.docx]

**Article title:** Understanding and measuring the work-related quality of life among those working in adult social care: A scoping review

**Journal name:** Health and Social Care in the Community

**Author names:** Barbora Silarova, Nadia Brookes, Sinead Palmer, Ann-Marie Towers, Shereen Hussein

**Affiliation and e-mail address of the corresponding author:** Barbora Silarova, PhD, Personal Social Services Research Unit, University of Kent, Cornwallis Central, Canterbury, CT2 7NF, UK

Email: B.Silarova@kent.ac.uk

**Online Resource 3** Description of key components of work-related quality of life (WRQoL) and items

| **Key components of WRQoL** | **Sub-themes** | **Items** |
| --- | --- | --- |
| **Organisational characteristics** | Working Culture | rewards, working conditions, environment, humour in the workplace, sufficient human and material resources, role ambiguity, role conflict, performing tasks that are conflicting or performing tasks one prefers not doing, physical, mental and emotional workload, time pressure, safety, decision latitude, clarity concerning a specific task or concerning the expectations of a client regarding a specific task, hours of work, forced overtime, job security, work procedures, rules, substitution during illness, working solo/opportunities for contact, communication with organisation, paperwork and  bureaucracy, protection of personnel's rights, economic well-being, ethics and diversity |
|  | Working Climate | feeling valued for individual's contribution, internal work motivation and general work fulfilment, empowerment, encouraging of new ideas and creativity, capacity for coping (social support from supervisors, colleagues, partner, clients, other caregivers; opportunities to discuss disagreements, being able to refer problems beyond the role to managers, relationships of trust), perceived influence on supervisors/managers, satisfaction with influence, staff positive contributions/positive experiences at work, the opportunity for social integration/interaction of personnel, the organisation’s commitment to quality and customers, influence in decision making |
| **Job characteristics** | Job–person match | staff empathy for people with challenging behaviour, challenging behaviour, self-efficacy, ‘Does your work demand a high level of skill or expertise?’, altruism, cognitive/emotional/physical demands and resources, encounters with death |
|  | Autonomy/Control at work | flexible framework, managing one's own work, latitude in decision-making,  resolution in problem-solving, being able to priorities care activities, control at work |
|  | Time | tasks taking up too much time and detracting from the more important functions of the job, which are considered to be spending time with and caring for the residents, time pressure and work demands, being pulled in too many different directions and juggling too many things during work day, time for appropriately performing nursing and care activities, constant changes, innovation overload, lack of time to adjust to new initiatives, ‘Have working conditions changed in recent years?’, possible changes in the work situation due to reorganisation, completely new work methods or similar |
|  | Responsibility for people | fear of ‘getting it wrong’, complex needs, increasing violence, abuse & behavioural problems, connection with clients |
|  | Learning and growth opportunities/self-actualisation | Development, growth in the organisation |
|  | Meaningful work | getting a lot out of working with the clients |
|  | Feedback from work | feelings of value and self-esteem, feeling inadequate, personal accomplishment, feeling of doing a good job, being appreciated for work |
| **Mental wellbeing and health** | Compassion satisfaction | subjective experience of happiness |
|  | Compassion fatigue | feeling emotionally and physically exhausted |
|  | Burnout/Work engagement | feeling irritable or frustrated while working, finding work overwhelming, ‘Have you experienced or ever felt close to burn out?’ |
|  | Mental well-being | general well-being, life satisfaction, subjective experience of happiness, ‘Do you believe care work has had an impact upon your mental health?’, ‘Do you believe care work has had a positive or negative impact upon your mental health?’, ‘Did you take any time off from work due to the impact of care work upon your own mental health?’, ’How much time did you take off of work due to the impact of care work upon your own mental health?’, ‘I am worried about my well-being/coping at work’ |
| **Physical wellbeing and health** |  | physical injury at work, time of work due to the work-related injury/injuries, ‘Have you sustained any significant physical injury at work?’, ‘How much time did you take off of work due to the injury/injuries you sustained whilst carrying out care work?’ |
| **Spill-over from work to home** |  | work-related thoughts when off duty, home–work interface, taking it home, work-life balance |
| **Professional Identity** |  | care workers see themselves as a professional, employer(s) respect care workers as a professional |

**Abbreviations:** WRQoL: work-related quality of life
